# A distinctive RhD serologic phenotype expressed by p.Glu233Lys RHD alleles DV type 5 and DAU4 in microplate agglutination RhD typing

**DOI:** 10.1111/trf.70408

**Published:** 2026-10-02

**Authors:** Vidyadhari Karne, Mark J. Jedrzejczak, Paul F. Lindholm, Glenn Ramsey, Jay P. Hudgins

**Affiliations:** ^1^ Department of Pathology Transfusion Medicine, Los Angeles General Medical Center/University of Southern California Los Angeles California USA; ^2^ Department of Pathology Feinberg School of Medicine, Northwestern University, and Blood Bank, Northwestern Memorial Hospital Chicago Illinois USA; ^3^ Department of Pathology and Laboratory Medicine Nationwide Children's Hospital Columbus Ohio USA

**Keywords:** blood group antigens, blood grouping and crossmatching, genotype, Rh blood group system

## Abstract

**Background:**

In patients with variant RhD phenotypes, serologic reactions for partial *RHD* genotypes are indistinguishable from other variants.

**Study Design and Methods:**

In two US transfusion services, patients were typed for RhD by automated *microplate RhD typing (Werfen,* Norcross, GA) using two directly agglutinating IgM anti‐D reagents, MS201 (epitope RhD (epD) 6.1) in Series 4 (Anti‐D4) and TH‐28 (epD6.4) in Series 5 (Anti‐D5). For weak or discrepant RhD typing, we performed a *RHD* DNA microarray (RHD BeadChip, Werfen, Norcross, GA). For hemi‐ or homozygous c.697G>A (rs1053359), we obtained c.1136C>T (rs61740966) testing (New York Blood Center) to distinguish DV type 5 (DV.5) from DAU4 (c.1136T). We retrospectively examined previous cases with negative‐weak anti‐D4 and strong anti‐D5 reactions for correlation with either DV.5 or DAU4 genotypes.

**Results:**

Eight of 442 patients with weak or discrepant RhD phenotypes (1.8%) had 0–1+ anti‐D4, 3–4+ anti‐D5 phenotypes. They were the only 8 patients with c.697G>A (correlation *p* < 0.0001, Fisher's exact test). Two had DV.5 (Hispanic, Armenian), 5 had DAU4 (4 Black, 1 Other), and 1 was not tested for c.1136 (Black). None had anti‐D.

**Discussion:**

DV.5 and DAU4 alleles express disruptive p.Glu233Lys and are associated with hemolytic anti‐D. *Microplate RhD typing* with decreased IgM anti‐D4 reactions did not associate DV.5 and DAU4 with alloimmunization but indicated increased risk of anti‐D alloimmunization. Similar epD6.1‐weak, epD6.4‐strong RhD reactions were observed in 10 DV.5 patients using automated microcolumn typing. Development of anti‐D reagents detecting patients with other partial *RHD* genotypes beyond DVI is desirable.

**Abbreviations**
D7B8: monoclonal IgM antibody clone for RhD antigen typing
DAU: RHD variant allele cluster
DBS: RHD variant allele cluster
DV: RHD variant allele cluster
DV.5: RHD allele DV type 5
D^W^ [Rh23]: low frequency antigen Rhesus blood group
EC3: extracellular loop 3
EC4: extracellular loop 4
EC5: extracellular loop 5
GC: Gamma‐clone
HDFN: hemolytic disease of the Fetus and Newborn
HTR: hemolytic transfusion reaction
MS201(D4): monoclonalIgM antibody clone for RhD antigen typing
RhD: Rhesus D antigen
RHD: Rhesus D gene
RUM‐1: monoclonal IgM antibody clone for RhD antigen typing
SCD: sickle cell disease
TH‐28 (D5): monoclonalIgM antibody clone for RhD antigen typing
Ѱ: RHD psuedogene

## 1 INTRODUCTION

In the United States (US), 0.1%–0.4% of patients have weak or discrepant RhD phenotypes using licensed directly agglutinating antisera.^1^, ^2^, ^3^, ^4^, ^5^, ^6^ Most such phenotypes arise from *RHD* gene variants which alter RhD expression. *RHD* genotyping is recommended to assess potential for RhD alloimmunization and guide need for RhD‐negative red blood cell (RBC) transfusions and Rh immunoprophylaxis. An expert US work group recommended that weak D types 1, 2, 3, 4.0, and 4.1 can be managed as wild‐type RhD positive.^7^ In the case of weak D type 4.0, some facilities may adopt more cautious management because alloanti‐D can occasionally develop, albeit with no fetal or transfusion hemolysis observed to date. For other variants with potential to make anti‐D, partial *RHD* with a recognized anti‐D risk and weak *RHD* antigens with uncertain anti‐D potential, RhD‐negative management is prudent. In two US national reference laboratories providing *RHD* genotyping (excluding conventional *RHD* and common D‐negative genotypes), 45%–50% of patients had weak D types 1, 2 or 3, 16%–18% had type 4.0, 22%–31% had other partial *RHD* genotypes and 6%–12% had other weak or uncommon variants.^8^, ^9^

Among weak or discrepant RhD phenotypes, antisera reactivity patterns are generally indistinguishable among variants managed as RhD+. Partial Rhd variants identified serologically, hence require *RHD* genotyping. Previously we reported experiences from Los Angeles and Chicago with genotyping patients identified as weak RhD, using an automated microplate RhD typing platform (Werfen, formerly Immucor) with two anti‐D reagents, Series 4 (Anti‐D4) and Series 5 (Anti‐D5).^1^, ^2^ Weak RhD phenotypes in this method are characteristically either weak in direct agglutination with one or both reagents or negative to weak only with anti‐D5. However, each institution's publications had an index case with weak anti‐D4, strong anti‐D5 reactions, and partial *RHD* allele DV type 5 (DV.5) or DAU4 were detected. We then collaborated on an investigation of subsequent anti‐D4‐weak, anti‐D5‐strong cases. We compared their phenotypes to those expressed by other DV and DAU alleles in the shared cohort. We also examined the role of anti‐D reagent clones employed in this platform and the implications for expanding transfusion safety serologically in patients with partial RhD phenotypic variants. This evaluation supports the hypothesis that *RHD* variant alleles *DV.5* and *DAU4* align with a specific serologic pattern of weak anti‐D4 and strong anti‐D5 reactivity with antisera for microtiterplate agglutination RhD typing.

## 2 METHODS

### 2.1 Serological methods

Test methods were described previously.^1^, ^2^ We performed routine RhD typing by automated *microplate RhD typing* via direct agglutination. Two monoclonal IgM anti‐D reagents were used for routine typing, MS201 in anti‐D4 and TH‐28 in anti‐D5. *RHD* genotyping was performed when: (1) either or both reactions were equivocal (Chicago) or ≤1+ (Los Angeles); (2) historical or current RhD typing were discrepant; or (3) RhD+ patients had anti‐D (Chicago). MS201 was historically assigned to RhD epitope (epD) 6.1.^10^, ^11^ TH‐28 is an updated term for the clone originally designated as NaTH28‐3C11 (data on file—Immucor, Inc.) (personal communication, Amanda Gallagher, June 5, 2025). NaTH28‐3C11 was assigned to epD6.4.^10^, ^11^ Some specimens also underwent RhD typing by tube direct agglutination with Gamma‐clone (GAMA401) (Chicago) or MS201 and TH‐28 (Los Angeles) (all tests: Werfen, Barcelona, Spain).

### 2.2 Genotyping and statistics

We performed *RHD* genotyping with a DNA microarray (RHD BeadChip, Immucor BioArray, Werfen, Norcross, GA). When hemi‐ or homozygous c.697G>A (rs1053359) was detected, DAU4 or DV type 5 was reported in BeadChip analysis by Basis software, and we obtained extended testing for *RHD* zygosity and the DAU marker c.1136C>T(rs61740966) in DAU4 (New York Blood Center). Two patients with other DV or DAU alleles observed in the phenotype comparisons had their *RHD* genotyping evaluated elsewhere (Versiti Blood Center, Milwaukee, WI) using RBC‐fluoGene D® weak/variant assay (Inno‐train Diagnostik GMBH, Clinton, NY). Statistical categorical comparisons employed Fisher's two‐tailed exact test for *p*< .05.

## 3 RESULTS

Among 442 total patients at both centers genotyped for weak or discrepant RhD phenotypes, 8 (1.8%) expressed a distinctive phenotype of weak anti‐D4 reactivity (0–1+) and strong anti‐D5 reactivity (3–4+) in *microplate RhD typing* (Table 1). These were also the only 8 cases with c.697G>A variants in DNA microarray testing (correlation *p*< .0001). They represented 6/237 cases in Los Angeles (2.5%) and 2/205 cases in Chicago (1.0%) (*p* = .3).

**TABLE 1:** Patients with 0–1+ D4(Anti‐D4) and 3–4+ D5(Anti‐D5) RhD typings in automated *microplate RhD typing*.

Case	Race‐ethnicity	Age‐gender	Diagnosis	D4/D5	Tube IS	RHD*
1^12^	Hispanic	23 F	Pregnancy 8 weeks	?/4+	3+ GC	*DV.5/01N.01*
2	Armenian	67 M	Leg burn	0/3+	1+ MS/3+ TH	*DV.5/01N.01*
3^2^, ^12^	Black	30 F	Pregnancy 9 weeks	?/4+	4+ GC	*DAU4/01N.01*
4	Black	46 M	Sickle cell disease	1+/3+	NA	*DAU4/01N.01*
5	Black	38 F	Pregnancy 7 weeks	1+/3+	NA	*DAU4/DAU4*
6	Black	24 F	Pregnancy 9 weeks	0/3+	NA	*DAU4/DAU4*
7	Other	27 M	Foreign body ingestion	?/4+	3+ MS/3+ TH	*DAU4/DAU4*
8^1^	Black	49 M	IV drug use	0/4+	2+ MS/4+ TH	DAU4 or DV.5/D*Ѱ

*Note*: All reactions are by direct agglutination. Case 8: *RHD* c.1136C>T testing not done.

Abbreviations: F, female; GC, Gamma‐clone; IV, intravenous; IS, immediate spin; M, male; MS, MS201; NA, not available; TH, TH‐28.

Two patients (one in each center) had partial DV.5 and were Hispanic and Armenian, respectively. Five patients had DAU4; 4 were Black and one's race‐ethnicity was designated as Other. One patient was not tested for c.1136C>T and was Black. In automated typing, DAU4 homozygosity did not affect anti‐D4 reaction strength. Among 5 DV.5 or DAU4 specimens typed by tube immediate spin, 2 hemizygous RBCs typed 1–2+ with anti‐D4; all other reactions were 3–4+. No patients had anti‐D. Four patients in early pregnancy subsequently received Rh immunoprophylaxis.

Table 2 compares RhD phenotypes for DV.5 and DAU4 cases with those expressed by 7 patients with other DV or DAU genotypes in the cohort. Phenotypes of the other DV or DAU patients were disparate but distinct from DV.5 and DAU4.

**TABLE 2:** RhD phenotypes expressed by other *RHD* DV and DAU alleles compared to DV.5 and DAU4.^1^, ^2^

Genotype RHD*	N cases	D4	D5	Tube
*DV.5*	2	0‐?	3–4+	
*DV.2* ^a^	1	4+	0	3+ GC
*DV.7 or DV.2 or DBS1* ^1^	1	0‐?	0	1+ D4/0 D5
*DAU4*	5	0–1+	3–4+	
*DAU2* ^1^	2	0‐?	?‐1+	2–4+ D4/1–2+ D5
*DAU3*	1	4+	?	3 + mf GC
*DAU3 or DAU11* ^a^	1	4+	4+	4+ GC
*DAU5 or DV.1 or DBS2*	1	4+	4+	4+ GC

*Note*: RHD*DV.7 or DV.2 or DBS1: RHD BeadChip readout. European ancestry: possibly DV.7.^13^ RHD*DAU3 or DAU11: Genotyped due to anti‐D. RHD*DAU5 or DV.1 or DBS2: RHD BeadChip readout. African ancestry and Dce phenotype: likely DAU5.^13^ Genotyped due to RhD typing discrepancy: previous RhD‐negative typing (method unknown) and Rh immune globulin therapy in Ghana. D4 and D5: anti‐D clones MS201 and TH‐28. GC: Gamma‐clone. mf: mixed‐field.

^a^ Genotyped at Versiti Blood Center, Milwaukee, WI.

## 4 DISCUSSION

### 4.1 DV.5 and DAU4

DV.5 and DAU4 have generally different racial and ethnic settings (Table 3). DAU4 is associated with African ancestry and Dce haplotype. An estimated 0.07% of US Blacks are hemi‐ or homozygous for DAU4.^14^ DV.5 was discovered in east Asia^15^, ^16^, ^17^, ^18^, ^19^ and subsequently found in Iran,^20^ Europe,^21^, ^22^, ^23^, ^24^, ^25^ Canada^26^, ^27^ and Australia,^28^ usually in DCe.

**TABLE 3:** Partial *RHD* DV type 5 and DAU4.

Characteristic	DV type 5	DAU4	References
Other names	DHK, DYO	None	[29]
Alterations	c.697G>A, p.Glu233Lys	c.697G>A, p.Glu233Lys c.1136C>T, p.Thr379Met	[29]
Usual Rh haplotype	DCe	Dce	[13]
RhD phenotyping	D^w^(−) (unlike other DV)	D^w^(−)	[30]
	Shared RhD variant monoclonal panel reaction profile		[31]
RhD antigen density^a^	NA	1909	[29]
Ancestry	European, SW Asia, E Asia	African	See text
*RHD* c.697G>A genetic ancestry allele frequencies (AF)^b^	European 0.0002% Admixed American 0.007% African/African‐American 0.08%		[14, 32]
AF, US Blacks/SCD	0%	0.13%	[14]
p. Glu233Lys structure	Predicted EC4 p.233 interaction shifted from EC5 to EC3		[33]
Hemolytic anti‐D	Mild HDFN case	HTR in SCD case	[21, 34]

Abbreviations: EC, extracellular loop; HDFN, hemolytic disease of fetus and newborn; HTR, hemolytic transfusion reaction; NA, not available; SCD, sickle cell disease.

^a^ Conventional RhD Dce antigen density 12,000–23,200 RhD/RBC.^35^

^b^ Allele frequencies in Genome Aggregation Database (gnomAD) version 4.1.0 were corrected for hemizygosity overcounting.^14^ Admixed Americans were formerly termed Hispanic/Latino in gnomAD.

The p.233 site in RhD extracellular loop 4 (EC4) is at an antigenically significant position. In all other DV alleles except DV.5, the *RHCE* conversion variant c.697G>C predicts p.Glu233Gln and expresses D^W^ (Rh23). However, DV.5 has c.697G>A predicting p.Glu233Lys. DAU4 has c.697G>A plus the DAU marker c.1136T predicting p.Thr379Met. DV.5 and DAU4 share a D^W^‐negative phenotype and the same reaction pattern in a panel of 12 monoclonal antibodies used for identifying RhD variants.^31^ This is consistent with the correlation value for the c.697G>A (correlation *p*< .0001, Fisher's exact test) mutation as the likely cause of the associated serologic findings. The presence of c.1136C>T mutation is associated with DAU cluster, and within that cluster the only DAU4 also contains the c697G>A mutation. In the RhesusBase (*RHD* allele database), Wagner retained the original allele name RHD*DHK for c.697G>A because of its different phenotype from other DV alleles.^36^

Structural modeling of the p.Glu233Lys protein predicted a notable conformational shift in intra‐protein interactions from p.286 on EC5 to p.164 on EC3.^33^ c.697G>A caused the only such predicted interaction switch from one EC loop to another among 42 single nucleotide variants analyzed by Floch et al.^33^ This may help account for the association of p.Glu233Lys with clinically significant anti‐D. Anti‐D in a mother with DV.5 caused mild hemolytic disease of the fetus and newborn.^21^ (This patient was published under the serological term DVa, but she had DV.5 as later reported for other *RHD* variant patients.)^23^ (personal communication, Jelena Lucacevic Krstic, Aug 2023). Anti‐D in a DAU4 patient with sickle cell disease caused a hemolytic transfusion reaction.^34^

### 4.2 Reagent anti‐D clones

To address the possible role of reagent anti‐D clones in the distinctive *microplate RhD typing* phenotype reported here, we sought all previously published direct agglutination reactions to DV.5 and DAU4 RBCs by IgM anti‐D antibodies classified as epD6.1 or epD6.4.^10^, ^11^ Data analysis from previously published DV.5 and DAU4 cases showed that one or more clones in each class gave negative or weak reactions with epD6.1 antibodies and positive or strong reactions with epD6.4 antibodies (Table S1, supplemental material online). An automated microcolumn RhD typing system employed by Lucacevic Krstic et al. gave weak ep6.1 reactions (clone RUM‐1) and strong ep6.4 reactions (D7B8) in 10 DV.5 patients.^23^ However, variability was seen across RBC cases, clones, and test methods.

### 4.3 Serological screening for partial *RHD*

Patients with partial *RHD* DVI have been protected from alloimmunization for over 20 years by using anti‐D reagents typing them as D‐negative in direct agglutination.^37^, ^38^, ^39^ In contrast, only 11% of US Blacks with partial *RHD* genotypes are recognized through weak or discrepant phenotypes in routine RhD typing.^14^ Some of these patients type as D‐negative and are managed accordingly, but many manifest a typical RhD+ phenotype and are not identified as candidates for Rh immune globulin or RhD‐negative RBCs. In sickle cell disease, 18%–20% of partial‐*RHD* patients form anti‐D.^6^ Development of RhD typing reagents to better identify patients with partial RhD phenotype has been proposed.^14^, ^37^, ^38^, ^40^, ^41^, ^42^

Based upon available literature, DV.5 was not previously reported in the US until detection via published data evaluating *microplate RhD typing*.^12^ The 5 DAU4 cases are a substantial number for a hospital‐based phenotyping study. DV.5 and DAU4 patients with partial *RHD* phenotype at risk for hemolytic anti‐D alloimmunization benefit from the weak anti‐D4 RhD phenotype we observed in US‐licensed microplate RhD typing. The association of weak anti‐D4/strong anti‐D5 with RHD alleles associated with a partial D phenotype and risk of anti‐D alloimmunization could be harnessed to develop reagents or approaches to screen for or omit from RhD antigen variant detection.^14^

### 4.4 Study limitations

Identified instances of DV.5 and DAU4 cases within the cohort are very infrequent. The low prevalence could represent low prevalence of these alleles or underpowered results. Further statistical analysis by blood collection facilities would widen the cohort size significantly, likely increasing the probability of confirming the finding from these institutions. Additional experience with those variants and with weak anti‐D4, strong anti‐D5 *microplate RhD typing could* confirm these findings. *RHD* genotyping is still needed for all patients with weak or discrepant RhD phenotypes for best determination of RhD management.

### 4.5 Conclusions

In two US hospitals, all patients with a distinctive phenotype of anti‐D4‐weak/anti‐D5‐strong reactions in routine licensed microplate RhD typing only express p.Glu233Lys partial‐*RHD* variants DV.5 or DAU4 in *RHD* genotyping. Development of more licensed RhD typing reagents to identify patients with other partial *RHD* genotypes beyond partial DVI would enhance patient safety.

## CONFLICT OF INTEREST STATEMENT

Glenn Ramsey is a consultant for Werfen.

## Supporting information


**Table S1.** DV type 5 and DAU4 RBCs: direct agglutination by IgM anti‐D clones classified in epitope patterns epD6.1 or epD6.4.

## Data Availability

Research data are not shared.
