# Supplementary material for: A distinctive RhD serologic phenotype expressed by p.Glu233Lys RHD alleles DV type 5 and DAU4 in microplate agglutination RhD typing
Source: Transfusion. 2026 Oct 2;66(11):2116–22. doi: 10.1111/trf.70408 (PMC13753057; doi:10.1111/trf.70408)
Supplement: Supplementary file 1 [file TRF-66-2116-s001.docx]

**Table S1.** DV type 5 and DAU4 RBCs: direct agglutination by IgM anti-D clones classified in epitope patterns epD6.1 or epD6.4

| **Allele - Clone** | **Reference** | **Cases** | **MAb** | **Test** | **Reactions** |
| --- | --- | --- | --- | --- | --- |
| DV.5 – 6.1 | Workshop^a^ 11 | DVa5, n? | 5 MAbs | Gel | Pos |
|  |  | DYO, n? | 5 MAbs | Gel | Pos |
|  | This study | 2 | MS201 | Auto plate | Neg-Eq |
|  |  | 1 of the 2 | MS201 | Tube | 1+ |
|  | 30 Krstic 2018 | 10 | RUM-1 | Auto gel | Weak-2+ |
|  |  |  | RUM-1 | Tube | Neg |
|  |  |  | 175-2 | Tube | Pos |
|  |  |  | P3*61 | Tube | 5 neg, 5 pos |
|  | 24 Omi | 2 | RUM-1 | NA | Neg |
|  |  |  | OSK3-2 | NA | Neg |
|  |  |  | P3*61 | NA | Pos |
|  | 26 Ye | 1 | P3*61 | Tube | Neg |
|  | 33 Denomme | 1 | 175-2 | Tube | Pos |
|  | 20 Krstic 2016 | 2 | MS201 | Tube | Pos |
|  |  |  | 175-2 | Tube | Pos |
|  | Clone summary except workshop: 15 of 18 cases neg-2+ with ≥1 MAb | | | | |
| DV.5 – 6.4 | Workshop^a^ 11 | DVa5, n? | 19 Mabs | Gel | Pos |
|  |  | DYO, n? | 19 Mabs | Gel | Variable |
|  | This study | 2 | TH28 | Auto plate | 4+ |
|  |  | 1 of the 2 | TH28 | Tube | 3+ |
|  | 30 Krstic 2018 | 10 | D7B8 | Auto gel | 4+ |
|  |  |  | TH28 | Tube | Pos |
|  | 25 Hyodo | 3 | B9A4B2 | NA | Pos |
|  | 28 Oodi | 1 | TH28 | NA | Neg |
|  | Clone summary except workshop: 15 of 16 cases 3-4+ or pos with ≥1 MAb | | | | |
| DAU4 – 6.1 | 15 Wagner 2002 | 1 | RUM-1 | Tube | Neg |
|  |  |  | P3*61 | Gel | Neg |
|  |  |  | 175-2 | Gel | Neg |
|  |  |  | MS201 | Gel | Pos |
|  | This study | 6 | MS201 | Auto plate | 0-1+ |
|  |  | 1 of the 6 | MS201 | Tube | 2+ |
|  |  | 1 of the 6 | MS201 | Tube | 3+ |
|  | 37 Rodrigues | 2 | MS201 | Tube | Weak+ |
|  | Person^b^ [new ref] | 3 | RUM-1 | Tube | 2 of 3 0-1+ |
|  |  |  | MS201 | Tube & plate | 1 of 3 0-1+ |
|  |  |  | 175-2 | Plate | 0 of 3 0-1+ |
|  | 4 Luo | 2 | MS201 | Auto gel | 3+ |
|  | 33 Denomme | 1 | 175-2 | Tube | Pos |
|  | Clone summary: 11 of 15 cases neg-1+ with ≥1 MAb | | | | |
| DAU4 – 6.4 | 15 Wagner 2002 | 1 | BS232 | Tube | Pos |
|  |  |  | BS226^c^ | Tube | Pos |
|  |  |  | NaTH28-3C11 | Gel | Pos |
|  |  |  | 7 other MAbs | Gel | Neg |
|  | This study | 6 | TH28 | Auto plate | 3-4+ |
|  |  | 2 of the 6 | TH28 | Tube | 3-4+ |
|  | Person^b^ [new ref] | 3 | TH28 | Auto plate | 2-4+ |
|  | 28 Oodi | 1 | TH28 | Tube | Neg |
|  | Clone summary: 10 of 11 cases 2-4+ or pos with ≥1 MAb | | | | |

^a^Workshop: All monoclonal antibodies (MAbs) named here were in the 2002 Paris workshop epitope classification except BS226 below. DVa5 and DYO are now both considered DV.5. Workshop reaction grades were not reported; reactions ≥25% of maximum were called positive (e.g., ≥1+ out of 4).^11^

^b^Person et al tested 3 DAU4 cases with 8 epD6.1 and 3 TH28 typing methods.[new ref below]

^c^BS226 was classified in 2001 by Wagner et al^38^ in epD17, which was renamed epD6.4 in 2002.^11^

Auto:

automated. Plate: microplate. Eq: equivocal. Neg: negative. Pos: positive.

Person RDM, Arnoni CP, Muniz JG, et al. Serologic strategy in detecting *RHD* altered alleles in Brazilian blood donors. Hematol Transfus Cell Ther 2020;42:365-72.
